# Electronic Energy Meter Based on a Tunnel Magnetoresistive Effect (TMR) Current Sensor

**DOI:** 10.3390/ma10101134

**Published:** 2017-09-26

**Authors:** Enrique García Vidal, Diego Ramírez Muñoz, Sergio Iván Ravelo Arias, Jaime Sánchez Moreno, Susana Cardoso, Ricardo Ferreira, Paulo Freitas

**Affiliations:** 1 Department of Electronics Engineering, University of Valencia, 46010 Valencia, Spain; engarvi@alumni.uv.es (E.G.V.); sergio.ravelo@uv.es (S.I.R.A.); jaime.sanchez@uv.es (J.S.M.); 2 INESC Microsystems and Nanotechnologies (INESC-MN), Institute for Nanosciences and Nanotechnologies, 1000-029 Lisbon, Portugal; scardoso@inesc-mn.pt (S.C.); pfreitas@inesc-mn.pt (P.F.); 3 Instituto Superior Técnico (IST), Universidade de Lisboa, Av. Rovisco Pais, 1000-029 Lisbon, Portugal; 4 International Iberian Nanotechnology Laboratory (INL), Av. Mestre José Veiga, 4715-31 Braga, Portugal; ricardo.ferreira@inl.int

**Keywords:** tunnel magnetoresistance, current sensor, energy meter, power measurement, wattmeter, internet-of-things

## Abstract

In the present work, the design and microfabrication of a tunneling magnetoresistance (TMR) electrical current sensor is presented. After its physical and electrical characterization, a wattmeter is developed to determine the active power delivered to a load from the AC 50/60 Hz mains line. Experimental results are shown up to 1000 W of power load. A relative uncertainty of less than 1.5% with resistive load and less than 1% with capacitive load was obtained. The described application is an example of how TMR sensing technology can play a relevant role in the management and control of electrical energy.

## 1. Introduction

Tunneling magnetoresistance (TMR) sensing is the fourth generation of magnetoresistive magnetic sensing technology after galvanomagnetic, anisotropic (AMR), and giant (GMR) technologies. The galvanomagnetic effect was achieved in semiconductors having a thickness greater than their length [1]. Better sensitivities were achieved using semiconductor materials such as InSb with Te as the preferred dopant atom [2]. AMR sensing technology was based on materials derived from binary and tertiary alloys of Fe, Ni, and Co, such as permalloy, deposited over a Si substrate. With these structures, AMR-based sensors achieved 2–4% of magnetoresistance variation, requiring a specific Barber pole geometry to have linearity and periodic flipping pulses to stabilize the internal initial magnetization over time [3]. With GMR sensing technology, 70% of magnetoresistance relative variation was reached [4], although in the most applications the variation was between 4% and 25%, depending on the microfabrication technology. The GMR basic structure is composed of two layers of ferromagnetic material (Fe, Co, and Ni alloys) separated by a non-magnetic material (like Cu). In practice, in order to have high magnetoresistance variation, new magnetic multilayers structures were fabricated by repetition of the basic structure (unpinned sandwiches, antiferromagnetic multilayers, and spin valves) [5,6,7,8,9,10]. TMR sensing technology is based on the magnetoresistive effect that occurs in a magnetic tunnel junction (MTJ) structure where the modulation of its resistivity is due to the spin-dependent tunneling effect (SDT). Once this effect was observed and technologically developed at room temperature, the design of new TMR-based sensors was made possible [11,12,13]. An MTJ element is composed of two ferromagnetic layers separated by an isolation layer. Usually, in the sensing technology, one of the ferromagnetic layers has immobilized its magnetization state (pinned) due to the presence of an antiferromagnetic layer [14], whereas the magnetization state of the second ferromagnetic layer is free to vary with the external magnetic field. The isolation layer is made of oxides such as MgO or Al_2_O_3_ [15,16]. The layer thickness of TMR structures is between 0.1 and 100 nm, and it is easy to find more than 200% of magnetorresistance variation at room temperature [17]. For industrial applications, TMR sensors are implemented in a Wheatstone bridge topology. Each resistor of the bridge is made of a series association of 100–300 MTJ elements, for improved electrical robustness [18]. TMR technology offers sensors with improved features in sensibility, linearity, thermal stability, and low consumption with respect with previous AMR and GMR technologies [19].

In the design of power monitoring systems and energy meters, the current sensing technology methods have been addressed for the use of shunt techniques, commercial current transformer-based probes, or Rogowski coils [20,21,22]. Magnetoresistive (MR) sensors offer an interesting alternative in applications where power processing requirements need compact and simple solutions. An experimental setup to process power with an MR sensor was developed in Reference [23]. It measures active power in the order of various tenths of milliwatts requiring additional circuitry to satisfy the flipping coil requirements. The features of MR-based watt converters have been analyzed using non-Wheatstone type MR topologies [24,25], or based on the bridge topology but at the level of integrated circuit monitoring, although not for industrial applications [26]. Considering previous current measurement techniques, resistive current shunts have ohmic losses that generate heat and do not provide electrical isolation. Hall-effect-based sensors need high permeability materials such as ferrites and, although isolation is well-provided, they have high volume and weight [27,28]. MR technology has developed current sensors with higher sensitivities without the use of ferromagnetic cores. In current measurement applications, MR sensors offer interesting features like high sensitivity, wide frequency response, and the ability to be microfabricated in high volume. These properties encourage their application in power or energy meters with low cost and optimal electrical characteristics.

In the present work, the design and microfabrication of a TMR electrical current sensor is presented. After its physical and electrical characterization, a wattmeter is developed to determine the active power delivered to a load. The described application is an example of how TMR sensing technology can play a relevant role in the management and control of electrical energy.

Electrical utility companies are interested in managing energy in situations such as high voltage distribution, substations, and domestic or industrial consumers. The user also is interested in monitoring the energy consumption of a load applied at the home (washing machine, cooker, small motors,) through the use of smart mobile phones and sensor networks. On the other hand, renewable energies have an increasing role in the whole energy mix. Electrical current and energy processed and generated in their production areas (wind turbines, photovoltaic fields) and their storage zones (batteries, supercapacitors, etc.) need to be known. The car industry is seriously changing the concept of mobility with the development of electrical vehicles (EVs). The electrical current and energy measurement and control are the most important variables to determine for the service provider (battery chargers) or for the car manufacturer (energy control, monitoring and management). All of these are examples where TMR sensing technology can be applied in the development of new systems and instruments based on new material and structures.

## 2. Sensor Description

### 2.1. Design and Microfabrication

**Figure 1:**
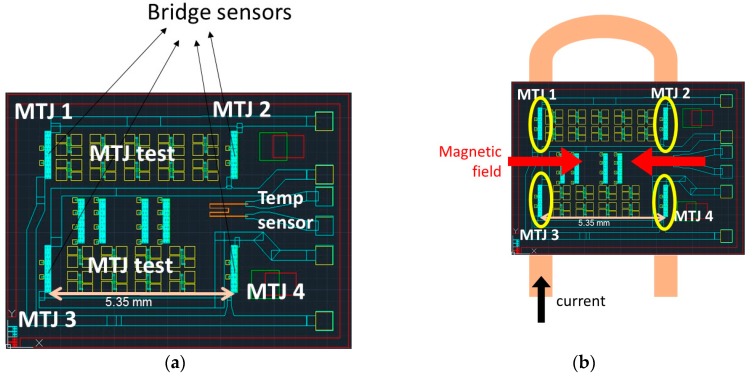
(**a**) Design of the chip with the four magnetic tunnel junction (MTJ) sensors connected in the Wheatstone bridge, several MTJ test elements, and a temperature sensor [30]; (**b**) Operation schematics, showing the opposite field created at each pair of MTJ elements by the U-shaped test current line.

The current sensor used in this work consists of a set of four magnetoresistive sensors (magnetic tunnel junctions, MTJs) implemented in a Wheatstone type topology (Figure 1a). The MTJ elements were deposited and fabricated simultaneously, and therefore have similar characteristic curves and reference layer orientations. The full bridge configuration was obtained so that the test conducting wire (U-shaped current line) mounted below the chip was aligned with each MTJ array (Figure 1b), to enable opposing magnetic fields in each pair of MTJ elements and opposite sensitivities to the magnetic field [29].

**Figure 2:**
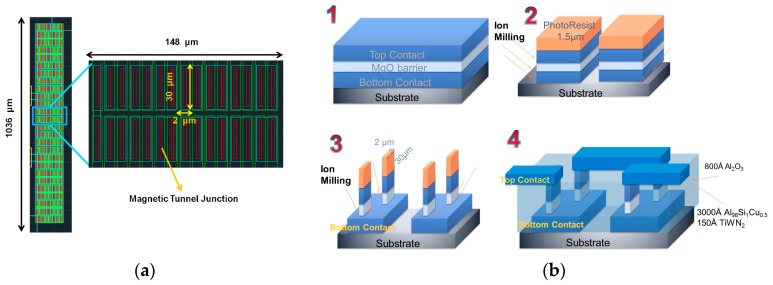
(**a**) Detail of the Wheatstone bridge resistances, which include 360 individual 2 × 30 µm^2^ MTJ elements connected in series; (**b**) Description of the microfabrication process to connect the 2 × 30 µm^2^ elements in series.

Figure 2a shows the detail of each of the four bridge resistances (MTJ-1, 2, 3, and 4). These are arrays of 360 MTJ elements (2 × 30 µm^2^) connected in series for enhanced electrical breakdown robustness [31]. The MTJ stack was deposited by sputtering in a Timaris tool [32], with the structure: Si/100 nm SiO2/5 Ta/15 Ru/5 Ta/15 Ru/5 Ta/5 Ru/20 IrMn/2 CoFe30/0.85 Ru/2.6 CoFe40B20/MgO 1/2 CoFe40B20/0.21 Ta/4 NiFe/0.20 Ru/6 IrMn/2 Ru/5 Ta/10 Ru (thickness in nm), showing a resistance area product of 22.6 kΩ.µm^2^. The four MTJ elements of the bridge were defined simultaneously, using optical lithography and ion beam milling in a Nordiko3000 tool, followed by a self-aligned process with an 80-nm-thick Al_2_O_3_ film. Top contacts consist of 300-nm-thick AlSiCu protected with 15 nm of TiW(N_2_) films, deposited by magnetron sputtering in a Nordiko7000 tool, and defined by lift-off. The process is described in Figure 2b.

### 2.2. Physical and Electrical Characterization

**Figure 3:**
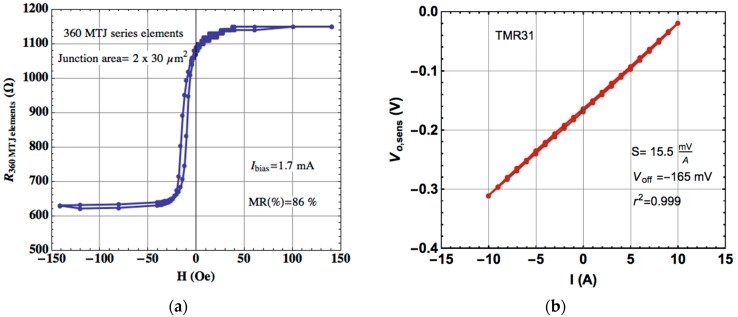
(**a**) R-H (resistance-to-field) characteristic of a series of 360 MTJ elements in association; (**b**) V-I (voltage-to-current) characteristic of the current sensor TMR31 (Wheatstone bridge) at room temperature.

The MTJ sensors were characterized individually, using the test structures indicated in Figure 1. Figure 3a shows the resistance-magnetic field characteristics of each MTJ sensor (360 MTJ elements in series), with magnetoresistance (TMR) values of 86%. The small coercivity and shift are the signature of the weekly pinned biasing at the free layer [31]. Figure 3b shows the bridge sensor output voltage under a current sweep between the interval −10 A to +10 A circulating through the U-shaped copper trace. A high electrical sensitivity of 15.5 mV/A was obtained, making the subsequent electronic signal amplification easier to achieve.

### 2.3. Practical Implementation

**Figure 4:**
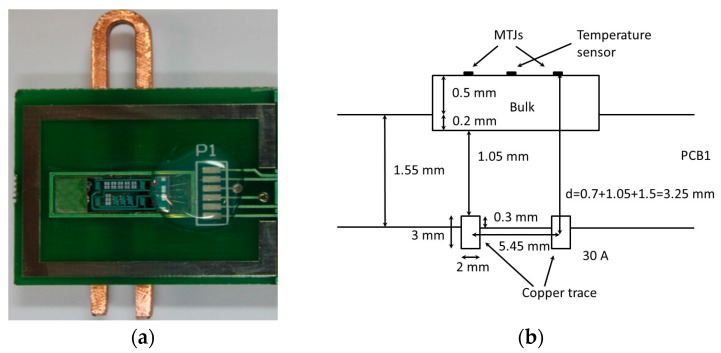
(**a**) Printed circuit board (PCB) including the complete tunneling magnetoresistance (TMR) transducer and the U-shaped copper current trace; (**b**) Cross-section schematics of the geometry used for this device, showing the copper trace dimensions as well as the sensor to trace distance.

The sensor was designed to measure up to 30 A of electrical current in a switched-mode power converter. A printed circuit board was constructed to mount the sensor, the conditioning electronics, and the copper conductor. The conductor was placed under the printed circuit board, separated an appropriate distance from the TMR elements in order to obtain the optimum linear range within their R-H characteristic (Figure 4).

## 3. Description of the Electronic Energy Meter

**Figure 5:**
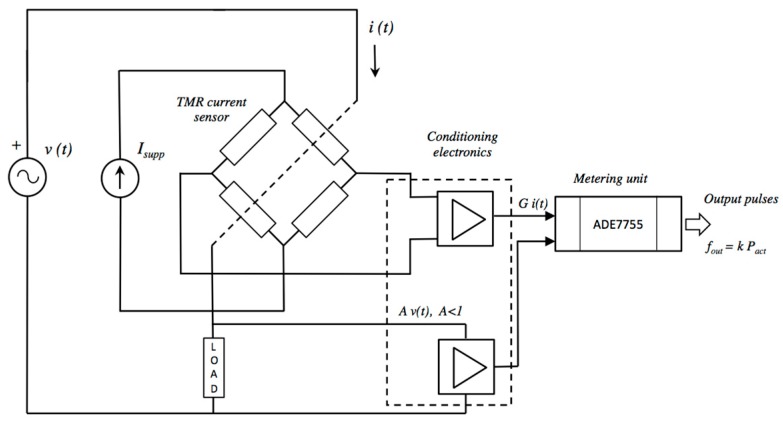
General block diagram of the TMR-based energy meter.

Figure 5 shows a general block diagram of the proposed electronic energy meter. It comprises the TMR sensor (described previously), the signal conditioning electronics, and the metering section.

### 3.1. Electronic Processing Unit (Metering Section)

**Figure 6:**
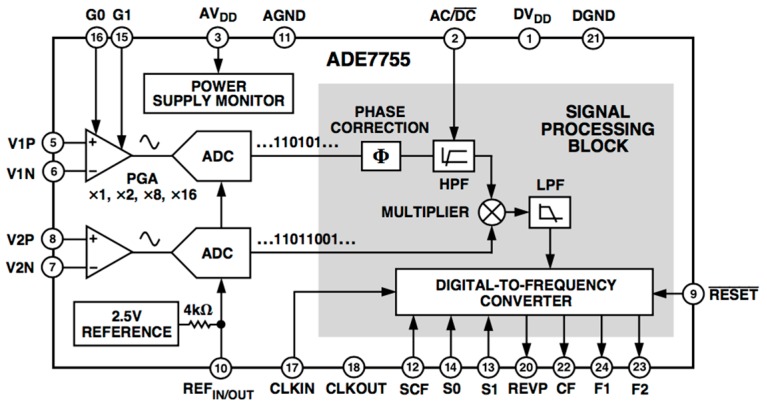
Internal block diagram of the ADE7755 active power processor [35].

There exists a wide range of electronic processors made by different manufacturers. They process electrical energy delivered to a load from the 50/60 Hz AC mains and offer at their output a variety of associated parameters like active and reactive powers or rms (root-mean-square) voltage and current [33,34]. In this work, the ADE7755 energy processor from Analog Devices (Norwood, MA, USA) was used [35]. This unit was designed to measure the active power supplied to an electric load by a monophasic AC line. Figure 6 shows its internal block diagram. It can be distinguished by three fundamental sections: analog signal processing and acquisition, digital signal processing, and conversion and power supply unit.

Analog signal processing and acquisition: It is composed of two programmable gain amplifiers (with values selected between ×1, ×2, ×8, and ×16). Each amplifier is used as the front-end of the voltage and current channels. The inputs of both amplifiers must be a low-level voltage amplitude and the line voltage and load current must be previously attenuated and conditioned. The output of each amplifier is acquired by an analog-to-digital (A/D) converter based on a sigma-delta conversion supplying 16-bit resolution.

Digital signal processing and conversion: It is the main block of the energy processor. As the central part, there is a multiplier that processes the digitalized voltage and current signals. The current channel has an additional phase correction block that compensates for the phase when the load has a strong inductive component, and it has a digital high-pass filter to reject possible offsets. The output of the multiplier is proportional to the instantaneous power delivered to the load and, once it has been low-pass filtered, a signal proportional to the active power is obtained. Finally, the digital-to-frequency converter outputs a signal with a frequency that indicates the active power and is prepared to interface easily with electromechanical or digital counters (energy registers).

Power supply unit: It is the part of the processor that provides the energy needed by the analog and digital units. Specifically, there is a 2.5 V precision reference voltage used by the A/D converters and, if needed, by the external conditioning electronics.

### 3.2. Signal Conditioning Electronics

**Figure 7:**
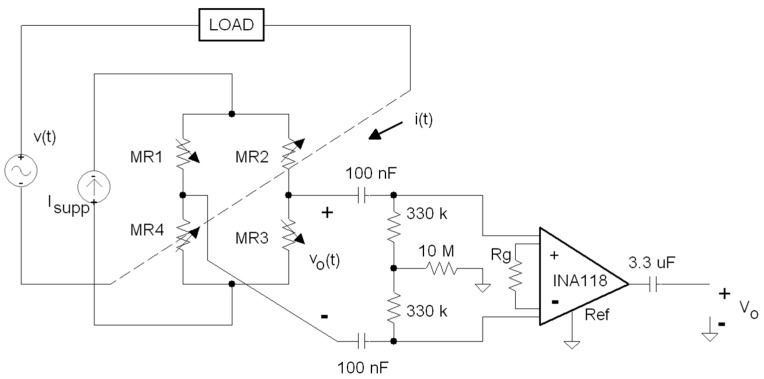
Signal conditioning electronics of the current channel.

Because the TMR current sensor is in a Wheatstone bridge topology, its output is a differential voltage signal v_o_(t) that requires differential-to-unipolar electronic conditioning. Figure 7 shows this electronic circuit in detail. A fully differential high-pass filter is connected at the current sensor output to reject DC offsets caused by the existent mismatching between the value of the four MR1 to MR4 active elements at zero current. Following the filtration, an instrumentation amplifier (INA118 model from Texas Instruments) is used to provide the necessary gain and to convert the differential signal in an unipolar V_o_ output voltage easily acquired by the energy processor. At its output, a simple 3.3 μF capacitor is connected to reject the possible offset due to instrumentation amplifier. I_supp_ is a DC constant that is the current source implemented to bias the Wheatstone bridge.

**Figure 8:**
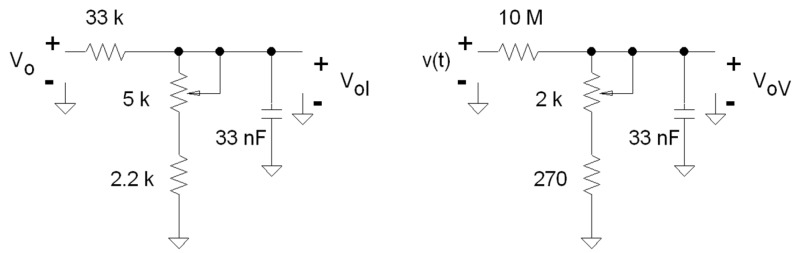
Voltage dividers and antialias filters for the current and voltage channels, respectively.

To adapt the voltage and current channels to the energy processor inputs, it is necessary to attenuate the output voltage of both channels. Both 250 mV_rms_ and 15 mV_rms_ voltages are present, respectively, at the current and voltage channel inputs of the ADE7755, using the values of the designed attenuators shown in Figure 8. The 33 nF capacitor prevents antialiasing for the time necessary for the processor to achieve the A/D conversion.

## 4. Experimental Results

**Figure 9:**
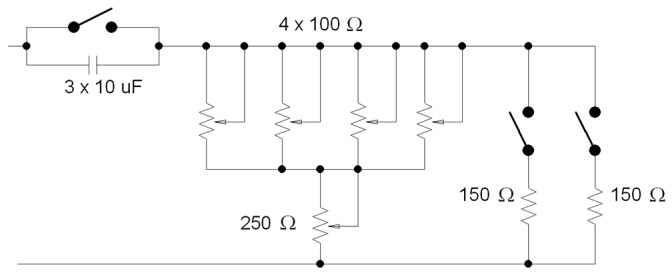
Variable load designed to test the TMR wattmeter.

After an adjustment process, the meter was submitted to load variations corresponding to different power consumptions. In the proposed wattmeter, a resistive variable load was built to change the power delivered to the load from 0 to 1000 W. Figure 9 schematically shows the variable load used to test the TMR wattmeter.

**Figure 10:**
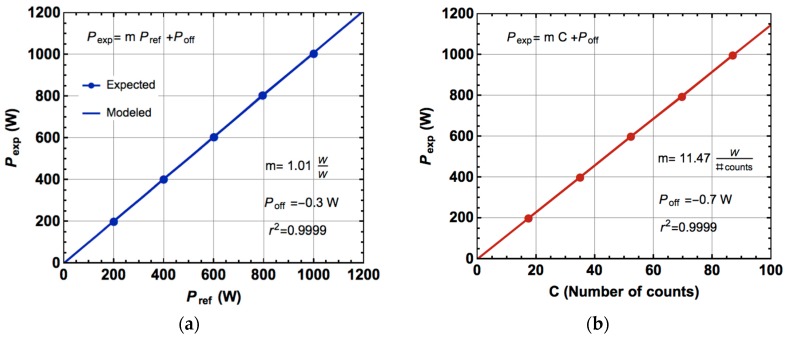

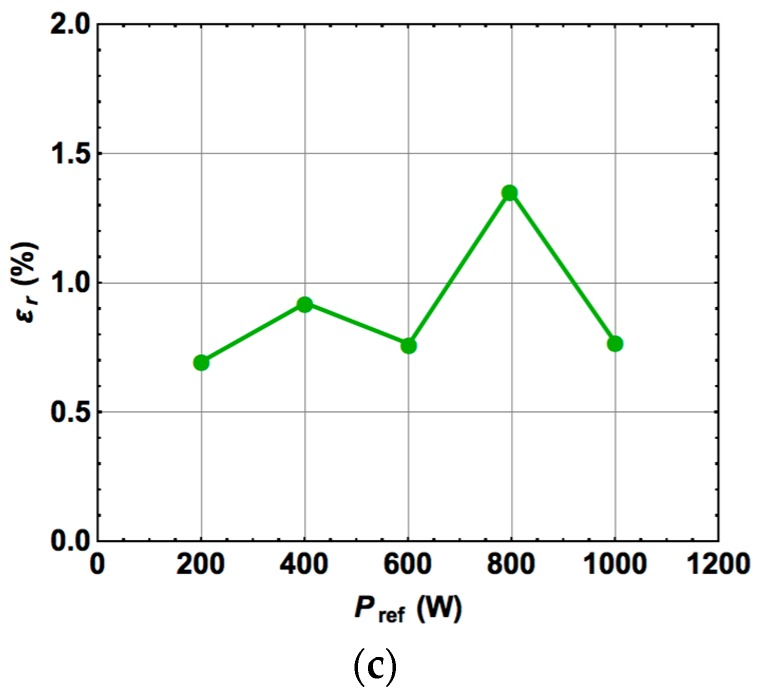
(**a**) Comparison between the experimental (TMR wattmeter) and reference wattmeter (2551 Xitron) active powers; (**b**) Actual active power vs. TMR wattmeter number of counts; (**c**) Obtained experimental relative uncertainty.

The experimental procedure was to take the number of output pulses provided by the ADE7755 at its CF (calibration frequency) output corresponding to 200, 400, 600, 800, and 1000 W power consumptions of pure resistive load during a 24-h period for each power value. A reference wattmeter (2551 from Xitron) was used to select the different powers delivered to the load. The experimental active power obtained from the CF output was compared with the readings of the reference wattmeter; Figure 10a shows that comparison and Figure 10b depicts the experimental relationship between the active power and the number of counts at the CF output. A conversion factor of 11.47 W/number of counts was obtained with a very low value of residual power (0.7 W). Figure 10c shows that the maximum relative uncertainty was less than 1.5% in the specified active power interval. To check the wattmeter behavior with no pure resistive loads, a 30 μF capacitor was connected in series (Figure 9) to shift the power factor to a cos ϕ = 0.75 value. Table 1 shows the relative uncertainty obtained with this test.

**Figure 11:**
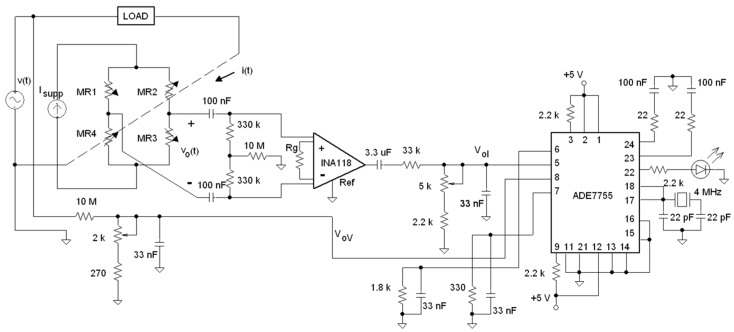
Whole schematic circuit that constitutes the TMR-based energy meter.

**Figure 12:**
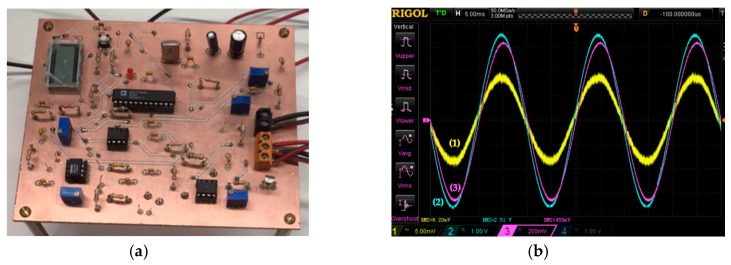
(**a**) Prototype energy meter implementation; (**b**) Experimental waveforms: (1) TMR sensor output voltage (6.2 mV_rms_); (2) Conditioned output voltage (2.51 V_rms_); (3) Electrical current through resistive load (4.59 A_rms_ and P_load_ = 1000 W).

As the final experimental results, Figure 11 shows the electronic schematic used to build the energy meter. That schematic was built in a practical prototype over a printed-circuit-board (PCB); Figure 12a shows such a practical implementation, and Figure 12b shows actual waveforms taken over the prototype.

## 5. Conclusions

An energy meter is presented based on a TMR current sensor with good linearity and relative uncertainty. The proposed work is applied to measure the active power delivered to a load from the AC 50/60 Hz main line. To measure current, conventional energy meters are based on the resistive-shunt method or Hall current sensors. The shunt method does not provide electrical isolation and has some self-heating properties due to the power dissipation on it. The Hall method provides electrical isolation from the mains but the use of ferrite cores causes bulky and heavy meters. The TMR method provides both electrical isolation and low volume, showing the feasibility to design new TMR-based instruments. The wattmeter presented is a prototype based on a microfabricated TMR sensor combined with a PCB, but an actual proof of concept can be built by integrating the sensor and the electronics in the same package or PCB. The presented work is an example of how spintronic-based materials and electronics engineering can produce new electronic measurement instruments. Both disciplines converge in a new one that could be named spintronics engineering.

## Figures and Tables

**Table 1:** Active power comparison with capacitive load.

Load	P_ref_ (W)	P_exp_ (W)	ε_r_(%)
R, 137.5 Ω	587.6	584.9	0.5
C, 30 μF			
cos ϕ = 0.75			
